# Topiramate versus naltrexone for alcohol use disorder: study protocol for a genotype-stratified, double-blind randomised controlled trial (TOP study)

**DOI:** 10.1186/s13063-018-2824-z

**Published:** 2018-08-16

**Authors:** Kirsten C. Morley, Henry R. Kranzler, Natasha Luquin, Andrew Baillie, Marian Shanahan, Ronald Trent, Maree Teesson, Paul S. Haber

**Affiliations:** 1 University of Sydney, Faculty of Medicine and Health, Central Clinical School, NHMRC Centre of Research Excellence in Mental Health and Substance Use, Sydney, NSW Australia; 2 Center for Studies of Addiction, Perelman School of Medicine, University of Pennsylvania and Mental Illness Research, Education, and Clinical Center, Crescenz VAMC, Philadelphia, PA USA; 3 Department of Medical Genomics, Royal Prince Alfred Hospital, Sydney, NSW Australia; 4 Faculty of Medicine and Health, Health Sciences NHMRC Centre of Research Excellence in Mental Health and Substance Use, University of Sydney, Sydney, NSW Australia; 5 National Drug and Alcohol Research Centre, UNSW, Sydney, NSW Australia; 6 NHMRC Centre of Research Excellence in Mental Health and Substance Use, National Drug and Alcohol Research Centre, UNSW, Sydney, NSW Australia; 7 Drug Health Services, Royal Prince Alfred Hospital, Sydney, NSW Australia

**Keywords:** Topiramate, Alcohol use disorder, Pharmacogenetics, Naltrexone, Alcohol dependence, GRIK1, OPRM1

## Abstract

**Background:**

Current treatments for alcohol use disorders have limited efficacy and there is a high degree of variability in treatment response. In a randomised, placebo-controlled clinical trial, there was a large effect size for topiramate in people homozygous for the *GRIK1* rs2832407*C allele. The primary aim of the TOP study is to examine prospectively the therapeutic and cost-effectiveness of topiramate versus an active control (naltrexone) in improving treatment outcomes for alcohol dependence. Participants will be stratified on rs2832407 to compare C-allele homozygotes with A-allele carriers to examine the moderating effect of rs2832407 on drinking outcomes. An exploratory aim is to examine the moderating effects of rs1799971, a polymorphism in *OPRM1*, on the response to naltrexone by comparing Asn40 homozygotes with Asp40 carriers.

**Methods/design:**

This double-blind trial will randomise 180 alcohol-dependent participants to a 12-week regime of either topiramate (titrating the dose up to 200 mg/day) or naltrexone (50 mg/day). Participants will be stratified on the two polymorphisms before randomisation. All participants will receive medical management. The primary drinking outcome will be the number of heavy drinking days per week and secondary drinking outcomes will include the time to relapse, the time to lapse and the percentage of abstinent days. Other secondary outcomes will include body mass index, tobacco use, anxiety symptoms and depressive symptoms.

**Discussion:**

If successful, the TOP study will improve management strategies for alcohol dependence by providing support for the use of genetic biomarkers to inform medication selection.

**Trial registration:**

ClinicalTrials.gov, NCT03479086. Registered on 27 March 2018.

**Electronic supplementary material:**

The online version of this article (10.1186/s13063-018-2824-z) contains supplementary material, which is available to authorized users.

## Background

Alcohol consumption is a leading cause of preventable death and is associated with over 200 diseases [1]. Although pharmacological treatment of alcohol use disorder (AUD) is now widely accepted, current treatments have modest efficacy and there is a high degree of variability in treatment response. Topiramate is increasingly being prescribed for AUD and its use now exceeds that of acamprosate, disulfiram and injectable naltrexone combined in the US veteran health system [2]. Topiramate antagonises glutamate activity at AMPA and kainate receptors [3], facilitates GABAergic function [4] and reduces the extracellular release of dopamine in the mesocorticolimbic region [5].

A recent meta-analysis of 1125 patients concluded that topiramate has a beneficial effect on heavy drinking and abstinence, with a somewhat larger effect size than naltrexone and acamprosate [6]. In alcohol-dependent patients, topiramate has also been observed to be efficacious in reducing body mass index (BMI) (by 1.2 kg/m^2^) relative to placebo [7], smoking rates [8] and symptom severity for anxiety and obsessive–compulsive disorder [9]. Effective interventions for psychological and physical conditions that are comorbid with AUD are particularly pertinent given their combined detrimental impact on the individual and the health-care system.

While a moderate signal of efficacy for topiramate in reducing heavy drinking has been demonstrated, there have been no well-controlled or well-powered direct comparative trials of topiramate with other commonly prescribed alcohol pharmacotherapies to guide treatment practice. Naltrexone was selected as the comparator for this trial because it is widely available as the standard of care for the pharmacotherapy of AUD [10]. One non-randomised, open-label trial showed that topiramate (200 mg/day) reduced alcohol intake and cravings significantly more than naltrexone [11]. The only randomised controlled trial (RCT) of topiramate versus naltrexone showed a non-significant trend towards a superior effect of topiramate, but the sample was small (*n* = 28) [12]. In addition, there have been very few economic evaluations of commonly prescribed medications for AUD [13, 14]. This absence of information on costs and potential economic benefits of treatment, given the significant burden of alcohol dependence, limits informed decision making by clinicians and policymakers.

The development of a personalised approach to treatment is an opportunity to improve the cost-effectiveness of pharmacotherapy by avoiding medications in patient groups unlikely to benefit or likely to experience adverse events. The heterogeneity in the effectiveness of alcohol pharmacotherapy may be related to variation in the genes encoding proteins involved in the specific mechanism of action of different drugs. Topiramate selectively acts on AMPA/kainate receptors via the GluK1 and GluK2 kainate subunits, which are encoded by genes *GRIK1* and *GRIK2*, respectively [4]. Variation in *GRIK1* has been demonstrated to be associated with alcohol dependence whereby the C allele of the single nucleotide polymorphism (SNP) rs2832407 was significantly overrepresented in individuals with alcohol dependence [15]. This provided the basis for studies of rs2832407 as a moderator of the response to topiramate. A secondary analysis of this hypothesis in a sample of 51 heavy drinkers showed that rs2832407*C-allele homozygotes reported fewer adverse events when treated with topiramate but no effect on treatment outcome [16].

More recently, a RCT demonstrated a robust moderating effect of rs2832407 on the therapeutic response to topiramate in a planned retrospective analysis [17]. This 12-week study demonstrated that patients treated with topiramate (with the dosage gradually increased from 25 mg/day to 200 mg/day) experienced fewer heavy drinking days (HDDs) per week than placebo-treated participants and higher odds of an abstinent day during week 12 in the topiramate group (odds ratio 2.57). This study demonstrated a large treatment effect in the rs2832407*C-allele homozygotes but not in the A-allele carrier group. In CC individuals treated with topiramate, the number needed to treat based on the criterion of no HDDs in the last 4 weeks of treatment was 2.28 compared to the full sample number needed to treat of 5.29. Similarly, the number needed to harm based on the presence of any severe adverse event was 9.09 compared to the full sample number needed to harm of 7.35. Thus, not only did CC individuals benefit from topiramate treatment, they also tolerated it better than A-allele carriers. In a follow-up analysis, these authors observed a moderating effect of positive alcohol expectancies on the relationship between rs2832407 and topiramate during treatment [18]. The beneficial effect of topiramate on daily drinking in CC individuals was lessened on days when these participants reported higher levels of anticipated positive outcomes of drinking. It is important to note that, although the results from this trial are promising for personalised medicine, they are based on secondary analyses rather than participants being prospectively stratified on genotype. This does not guarantee equivalence of group size nor baseline characteristics that may moderate or mediate treatment outcome.

The majority of naltrexone pharmacogenetic studies have examined the role of rs1799971 (or Asn40Asp), a SNP in the gene encoding the mu-opioid receptor (*OPRM1*), as a moderator of treatment response. Most retrospective analyses have shown greater treatment efficacy in heavy drinkers with Asp40 who were treated with naltrexone [19, 20]. However, more recently, data from two prospective studies that oversampled the variant allele failed to show a moderating effect [21, 22]. These inconsistencies are most likely due to the sampling bias inherent in post hoc analyses of clinical trial data [21]. Prospective genotype-stratified trials are required to confirm retrospective observations from a secondary analysis of pharmacotherapy trials.

Accordingly, the current study aims to examine the treatment and cost-effectiveness of topiramate versus naltrexone in improving outcomes in active drinkers and prospectively to examine the moderating role of the rs2832407 polymorphism in *GRIK1* on treatment response. We hypothesise that:

i. Topiramate-treated patients will significantly reduce their drinking, as measured by the number of HDDs, compared to oral naltrexone.
ii. Topiramate treatment will be more cost-effective than oral naltrexone.
iii. rs2832407 will have a moderating effect on drinking outcomes in topiramate-treated patients.
iv. Comorbid clinical conditions (e.g. obesity, smoking rates and anxiety) will be significantly improved in topiramate-treated patients compared to those on naltrexone.

We will also conduct exploratory analyses of the moderating role of (i) the Asn40Asp SNP in *OPRM1* on drinking outcomes in naltrexone-treated patients and (ii) baseline clinical characteristics on treatment response to topiramate versus naltrexone. This article presents the protocol for the RCT and is written to comply with the recommended SPIRIT guidelines for RCT protocols (Additional file 1) [23].

## Methods/design

### Study objectives

We are conducting a 12-week, double-blind, randomised trial to examine the effectiveness of topiramate versus naltrexone in improving treatment outcomes for heavy drinkers.

### Patient recruitment

The trial is being conducted in several outpatient hospital settings across Sydney, NSW, Australia. We aim to recruit 180 participants by clinical referral from treating physicians, nurses and psychologists among inpatients and outpatients of participating hospitals. We will also recruit via flyers and community advertisements at local general practitioners, newspapers and websites. Based on our previous experience, recruitment from mixed sources does not significantly impact treatment retention or outcome in alcohol pharmacotherapy trials [24]. After detoxification according to local procedures as clinically indicated, consenting participants enter the study. Participant time and travel expenses are reimbursed at the follow-up research assessments.

### Randomisation and allocation concealment

The study is a double-blind, randomised trial with participants being randomised to receive one of two possible medication arms. The study is conducted under double-blind conditions so that participants and study staff are unaware of medication assignment. A computer-generated random allocation to topiramate or naltrexone is being provided by an independent service at the Clinical Trials Centre of the National Health and Medical Research Council (NHMRC) in Australia using minimisation stratified by *GRIK1* genotype, *OPRM1* genotype and the concurrent use of antidepressants. This is provided to the clinical trials pharmacist at each site for allocation to an intervention and dispensing medication. Researchers, clinicians and participants are all blinded to treatment allocation. In the event of a medical emergency that requires knowledge of the treatment condition in the opinion of the treating clinician at the time, the investigators can contact the 24-h telephone service at the NHMRC Clinical Trials Centre to break the randomisation code for that individual.

### Inclusion and exclusion criteria

#### Inclusion criteria

The inclusion criteria are:

1. Aged 18 to 70 years
2. AUD according to the DSM-5 criteria
3. Adequate cognition and English language skills to give valid consent and to complete research interviews
4. Average weekly alcohol consumption of ≥30 standard drinks for men and ≥25 standard drinks for women, with a weekly average of ≥2 HDDs during the month before screening
5. Written informed consent
6. Willingness to provide a blood sample for genotyping

#### Exclusion criteria

The exclusion criteria are:

1. Active major psychiatric disorder associated with psychosis, significant suicide risk, or signs of impaired cognitive functioning
2. Pregnancy or lactation; women are advised to use reliable contraception for the duration of drug therapy and a urine pregnancy test is performed as appropriate
3. Concurrent use of any psychotropic medication other than antidepressants (provided that these are taken at stable doses for at least 2 months)
4. Currently taking any tricyclic antidepressant, e.g. Adapin (doxepin), Anafranil (clomipramine), Elavil (amitryptyline), Pamelor (nortryptyline), Tofranil (imipramine) or Sinequan (doxepin)
5. Use of antiretroviral dolutegravir
6. Dependence on any substance other than nicotine
7. Opioid abuse, opioid dependence or opioid agonist treatment, or likely need for opioid treatment
8. Clinically decompensated liver disease (jaundice or other signs of liver failure)
9. History of nephrolithiasis
10. History of glaucoma
11. Lack of stable housing or contact phone number
12. Previous hypersensitivity to topiramate or naltrexone
13. Any alcohol pharmacotherapy within the past month

### Procedures and schedule of visits

**Fig. 1:**
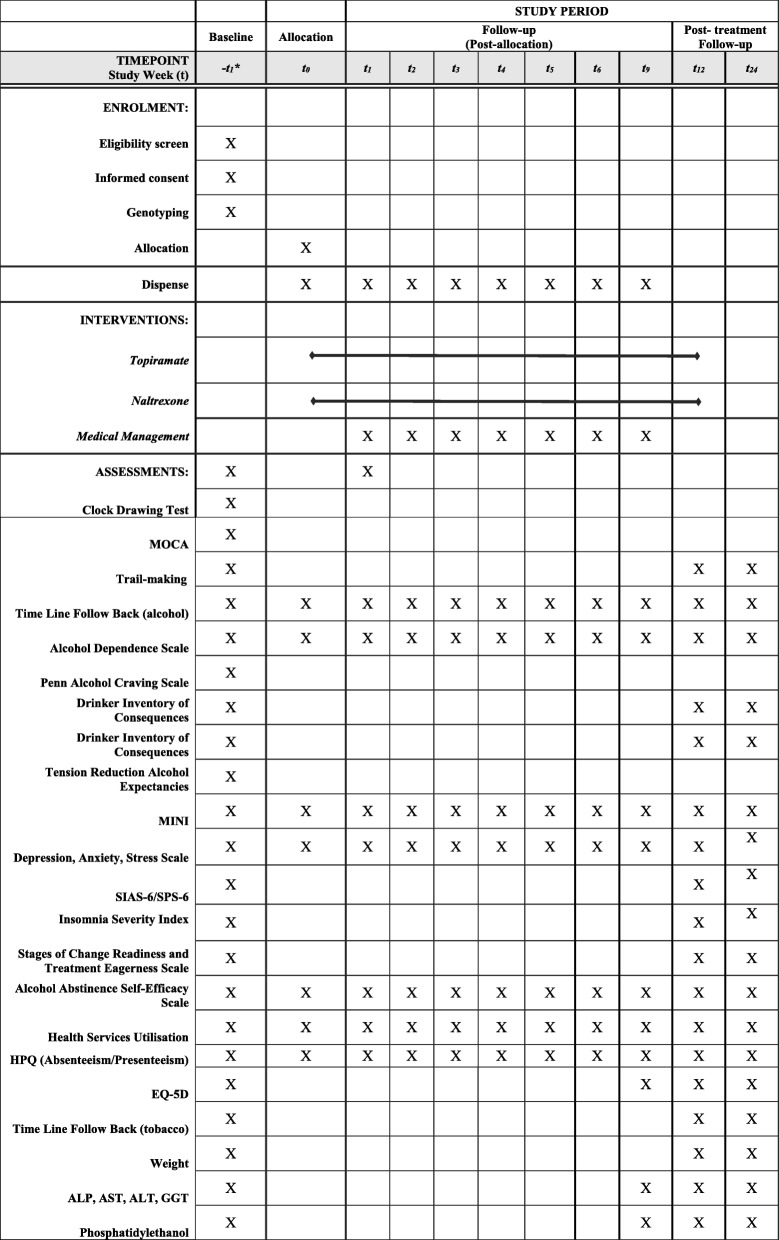
SPIRIT figure of the TOP study protocol.

Patients are initially assessed for suitability by a medical officer and offered withdrawal management as clinically indicated. Suitable patients are offered screening. The schedule of enrolment, interventions and assessments is depicted in Fig. 1.

#### Visit 1 (screening visit)

At the screening visit (visit 1), patients are asked to provide informed consent by the research nurse. The research nurse obtains a structured medical and psychiatric history. Blood and urine samples are taken for clinical laboratory evaluations (see below).

#### Visit 2a (baseline visit)

Eligible patients undergo a research evaluation (90–120 min) 2–7 days after screening, during which time genotyping and if necessary alcohol withdrawal management are completed. A study physician performs a physical examination.

#### Visits 2b to 10 (treatment and follow-up visits)

Patients receive initial counselling and study medication during a treatment session (2b) on the same day as the baseline visit (2a). Patients are given either naltrexone 50 mg/day or topiramate titrated up to a maximum of 200 mg/day by week 6 (see below for details). During the 12-week treatment period, patients return weekly for medical management and medication dispensing for the first 6 weeks and then every 3 weeks until the end of the treatment period. They return for the final follow-up medical review and research appointments at weeks 12 and 24.

### Pharmacotherapy schedule

#### Topiramate

The maximal dosage of topiramate is 200 mg/day in two divided doses based on prior evidence of efficacy, tolerability and high completion rates [12, 25]. Topiramate is registered as a prescription medication in Australia (Australian Register of Therapeutic Goods IDs 135797, 135790 and 135,766) and is purchased from Sandoz Pty Ltd. A gradual 6-week titration period that was well tolerated in previous studies is used [17]. The dose of topiramate is not increased if the previous dose is not well tolerated (in previous experience, for <5% patients), as indicated by the reporting of adverse events, the discretion of the physician or at the request of the participant. The nurse will provide guidance for patients on the titration schedule in consultation with the study physician. The schedule is

- Week 1: 25 mg afternoon
- Week 2: 50 mg afternoon
- Week 3: 75 mg (25 mg morning and 50 mg afternoon)
- Week 4: 100 mg (50 mg morning and 50 mg afternoon)
- Week 5: 150 mg (50 mg morning and 100 mg afternoon)
- Weeks 6–12: 200 mg (100 mg morning and 100 mg afternoon)

#### Naltrexone

The dosage of naltrexone is 50 mg daily. This group receives two capsules per day to match the topiramate dosage (placebo in the morning and naltrexone in the afternoon). Naltrexone is registered as a prescription medication in Australia (Australian Register of Therapeutic Goods ID 128710) and is purchased from Generic Health Pty Ltd.

Medications are over-encapsulated to maintain the double-blind and formulated in two different coloured capsules (morning versus afternoon doses). At the end of the 12-week treatment period, the participant and the researcher are asked to record the drug the participant is thought to have received. Limiting the dose (rather than titrating upwards) is permitted according to clinician judgement and is recorded to optimise treatment outcome by minimising side effects and maximising adherence. Participants who wish to cease medication are advised to withdraw medication gradually over 1 week. Any participants who wish ongoing treatment are offered open-label pharmacotherapy at their own expense subject to the judgement of their clinicians. Clinical data for any such individuals are being collected.

### Medical management

At each treatment visit, all patients receive 20–30 min of medical management, which was developed for the COMBINE trial as a medically oriented intervention to maximise medication adherence in the treatment of AUD [26]. Clinicians with minimal specialty training who are knowledgeable about AUD can deliver this brief, effective and widely used intervention. Medical management is, thus, feasible in all study sites and unlikely to obscure a medication effect on drinking outcomes. In medical management, the clinician highlights the participant’s symptoms related to problematic alcohol use and the need for treatment. The participant is advised to reduce or stop drinking, educated about alcohol-related disorders, given a rationale to take medication, instructed on the importance of medication adherence and given information on the medications. The research nurse also checks the participant’s breath alcohol level, vital signs and weight, medication adherence, drinking and smoking, and general functioning, and obtains a urine sample to measure medication adherence. Participants provide reports of side effects at each study visit. Patients are asked to return unused study medication at each treatment visit. Capsule counts and usage are recorded. Participants will be encouraged to defer concurrent psychotherapy until at least week 6 of the trial.

### Study assessments

#### Medical and laboratory tests

Medical and laboratory tests are to screen participants for exclusion criteria, assess potential adverse effects, provide objective measures of alcohol use, and assess covariates and secondary outcome measures. The physical examination at baseline includes a blood pressure and cardiovascular examination, checks for alcohol-related liver disease, a brief neurological examination and a mental state examination. Blood and urine are obtained at screening and at weeks 6, 12 and 24. Laboratory tests include: urinalysis, urine toxicology, full blood count, liver function tests (for bilirubin, gamma glutamyl transpeptidase, alkaline phosphatase, aspartate transaminase, alanine aminotransferase, albumin and protein), coagulation tests (international normalized ratio and activated partial thromboplastin time), creatinine and phosphatidylethanol. Bicarbonate and electrolytes are checked to screen for metabolic acidosis, an adverse effect of topiramate treatment that is readily reversible (weekly as required). Blood samples are also collected and stored for biochemical, genetic and molecular analysis in the current trial and for future use in ancillary studies.

### Psychological assessments

At baseline, demographics, medical history, personal and family history of AUD, and alcohol treatment history are obtained as in our previous trials [27–30]. Psychiatric diagnostic information is obtained with the MINI Neuropsychiatric interview [31]. The following measures are implemented at baseline and follow-up.

1. Characteristics of alcohol dependence (baseline and follow-up):
  (i) Recent (last 30 days) alcohol consumption (frequency and quantity) assessed by the timeline follow-back method (TLFB) [32], with which we also measure tobacco use
  (ii) Severity of alcohol dependence assessed by the Alcohol Dependence Scale [33]
  (iii) Craving for alcohol measured by the Penn Alcohol Craving Scale [34]
  (iv) Drinker Inventory of Consequences (at baseline and at follow-ups) [35], which provides a measure of the social, physical and emotional consequences of alcohol use
  (v) Tension Reduction Alcohol Outcome Expectancies, which measures expectancies regarding the outcome of alcohol use [36].
2. Mental health:
  (i) Depression, Anxiety and Stress Scale, which measures the severity of symptoms of depression, anxiety and stress [37]
  (ii) Sleep problems assessed by the Insomnia Severity Index [38]
3. Motivation and self-efficacy:
  (i) Stages of Change Readiness and Treatment Eagerness Scale (SOCRATES) assesses motivation to change [39]
  (ii) Alcohol Abstinence Self-Efficacy (AASE) scale measures perceived self-efficacy [40]
4. Cognitive and executive function:
  (i) The Montreal Cognitive Assessment (MoCA), which is used to screen for cognitive impairment that is likely to preclude the completion of study procedures and treatment [41]
  (ii) Trail Making Test [42], which is used to screen for impairment and to measure the speed of processing and executive functioning
5. Physical health:
  (i) Health outcomes measured using the EQ-5D questionnaire [43] and Health and Performance Questionnaire (HPQ) [44]

We also collect daily drinking, medication usage and alcohol expectancies via SMS (automated email to SMS). Daily paper diaries supplement SMS where necessary. An assertive follow-up will be made to obtain data for all randomised participants, regardless of whether they drop out of the study.

### Data and safety monitoring

Adverse events are collected at every study visit and the site investigator rates their relationship to the medication. Serious adverse events are defined by the Therapeutics Goods Administration [45]. If serious or unexpected adverse events occur during the trial, they will be reviewed by the principal investigator and the trial team and be reported within the specified time to the lead hospital ethics review committee and to the data and safety monitoring board (DSMB), which comprises members independent of the research study and funding body. The DSMB will meet regularly to provide oversight and monitoring to ensure participants’ safety and the trial’s validity and integrity. The DSMB can request that the trial be terminated by the principal investigator in the event of safety concerns. Data will be double entered and source verification (data auditing) undertaken by a separate researcher upon the completion of the trial. Participants will be assigned a code and their information will be de-identified in password-protected documents.

### Primary outcome

The primary outcome is alcohol consumption expressed as self-reported number of HDDs (defined as at least four drinks for women and at least five drinks for men) per week. Any conflicting information will be validated by collateral reports and measurements of biological markers of alcohol consumption (phosphatidylethanol).

### Secondary outcomes

We will examine the time to lapse (any alcohol), time to relapse to heavy drinking, percentage number of days abstinent and the number of standard drinks per drinking day. We will also compare topiramate versus naltrexone on adverse events and BMI, tobacco use, levels of anxiety and depression (Depression, Anxiety and Stress Scale), cravings (Penn Alcohol Craving Scale), alcohol-dependence severity (Alcohol Dependence Scale), markers of liver injury (liver function tests) and sleep disturbances (Insomnia Severity Index and Pittsburgh Sleep Quality Index). We will explore the degree to which these clinical characteristics moderate treatment response and the mechanisms underlying these phenomena. We will also examine daily alcohol expectancies, drinking levels and the interaction of these factors with medication and genotype as in previous work [46].

### Genotyping

Genotyping of rs2832407 and rs1799971 will be performed rapidly (i.e. between the first and second visits) in the Department of Medical Genomics, Royal Prince Alfred Hospital, a recognised NATA-RCPA certified DNA genetic testing laboratory within NSW Health Pathology. DNA from whole blood will be isolated using a QIAcube automated extraction instrument (Qiagen). Genotyping for the SNPs rs2832407 in *GRIK1* and rs1799971 in *OPRM1* will be performed using TaqMan® SNP Genotyping Assays (Life Technologies). A polymerase chain reaction analysis will be performed using the following parameters: 60 °C for 30 s (data collection), 95 °C for 10 min, followed by 40 cycles of 95 °C for 15 s and 60 °C for 1 min (data collection), and finally 60 °C for 30 s (data collection). Genotypes will be read using the TaqMan Genotyping Software (v3.1) (Applied Biosystems). Any remaining whole blood will be stored for potential use in future studies.

### Sample size calculations

Power is considered for a two-sided test at α = 0.05 and a within-subject correlation of 0.35–0.40 for repeated measures. For the primary aim, the naltrexone versus topiramate analysis (grouped by time), we considered power for the comparison of the topiramate and naltrexone groups to have a small-to-moderate effect size, Cohen’s *d* = 0.33, at week 7 that will persist through week 12 based on Baltieri et al. [12]. Therefore, we have more than 80% power to detect a small effect size with our recruiting aim of *N* = 180 (*N* = 90 in each group). For the secondary aim, analysis of the moderating effect of *GRIK1*, we consider just the topiramate-treated patients in Kranzler et al. [17] in which the CC versus A carrier groups were separated at week 6 by a moderate effect size. Therefore, we have more than 80% power to detect a moderate effect size with *N* = 90 (as per above). The frequency of the rs2832407 C allele in individuals of European descent is approximately 60–65%, with 41–43% C allele homozygotes [15]. Our sample size calculations, thus, account for a prevalence of approximately 42% of the responsive CC genotype. The moderating effect of *OPRM1* is an exploratory aim given that: (i) there have been several investigations of the moderating role of this SNP on naltrexone in alcohol patients and (ii) the prevalence of Asp40 carriers is 15–25% and potential under-sampling will, thus, reduce the power to test the effect.

### Statistical analysis

All available data from participants who took at least one dose of medication will be used, following the intention-to-treat principle. The primary outcome measure is frequency of HDDs (at least five standard drinks per day for men and at least four for women), which is a sensitive clinically relevant measure for alcohol trials [47]. Secondary outcomes (in addition to those listed above) will include number of abstinent days and average drinks per day. The two medication groups (naltrexone versus topiramate) will first be compared on weekly HDDs using mixed effects models followed by an analysis of secondary outcomes. Additional covariates will be included in the sensitivity analyses. Contrasts will also examine the final 6 weeks of the treatment period. Categorical variables will be compared using a chi-squared test. The medication by genotype groups will be compared using mixed models with fixed effects as intervention group (topiramate or naltrexone) and genotype (rs2832407*CC versus A carrier) as a moderating variable. The topiramate medication group will also be separately analysed with genotype as the fixed effect (CC versus A carrier). As with all alcohol treatment studies, we anticipate some participant dropout and intermittent missing data. Participants will be followed irrespective of whether they continue to receive treatment. Mixed effects models make use of all available responses under an assumption of ignorable missingness. The impact of missing data on the primary outcome measure will be explored by employing multiple imputation techniques and sensitivity analyses will be conducted to measure the effect of missing data on reported outcomes.

### Cost-effectiveness analysis

The cost-effectiveness analysis will take a societal perspective. The primary outcome will be quality adjusted life years (QALY) measured by the EQ-5D [43], a standardised instrument for measuring health outcomes (collected at baseline and follow-up), which is widely used in general population research internationally. The EQ-5D will be administered at multiple time points and the area under the line of their curve will estimated [48]. We expect that, at least in the short term, there will be little or no demonstrated effect on QALY. For this reason, the primary clinical outcome, reduction in alcohol consumption, will be a secondary outcome in the cost-effectiveness analysis.

Detailed information on resources associated with the provision of the intervention (training of clinical staff, screening and assessment, diagnostic and genetic tests, and medication) will be obtained from each clinical site’s electronic clinical records. The use of other health-care resources will be collected through data linkage of emergency department and inpatient hospital records and by self-report using a modified health services utilisation data collection form. Hospital utilisation will be costed with appropriate unit cost weights. Other health-care resources (general practice visits, etc.) will be costed using unit costs obtained from a range of sources including State Wage Awards [49], the Pharmaceutical Benefits Schedule [50] and the Manual of Resource Items published by the Pharmaceutical Benefits Advisory Committee [50]. Lost productivity and personal costs will be collected by structured self-report (HPQ: Clinical Trials Version) [44]. The HPQ contains questions about absenteeism (hours and days missed from work), and the quality and quantity of work while at the job (often referred to as presenteeism). All costs, including any related to adverse events, will be summed and combined with the outcome measure, and the incremental cost-effectiveness ratio:

$$ \mathrm{ICER}=\left(\mathrm{Cost}\ \mathrm{of}\ \mathrm{topiramate}-\mathrm{Cost}\ \mathrm{of}\ \mathrm{naltrexone}\right)\times N\Big)/\left(\mathrm{Eff}\ \mathrm{topiramate}-\mathrm{Eff}\ \mathrm{naltrexone}\right). $$

Bootstrapping will be conducted to obtain reliable confidence intervals from skewed data, and cost-effectiveness acceptability curves calculated. Our analysis will account for any differences in sample groups if necessary.

## Discussion

The TOP study will examine the therapeutic and cost-effectiveness of topiramate versus naltrexone in improving alcohol outcomes in active drinkers and will prospectively examine the moderating role of the rs2832407 polymorphism in *GRIK1* on treatment response. The trial will also examine the effectiveness of topiramate versus naltrexone on a range of comorbid conditions associated with AUD, including obesity, tobacco use and anxiety symptoms. It is critical for genetic association research to be both replicated and to be conducted prospectively to reduce the likelihood that observed associations were not due to type 1 errors. Similarly, prospective reporting of this clinical trial protocol contributes to clinical trial quality [51]. This report has been prepared in accordance with the SPIRIT protocol for reporting of clinical trial protocols [23].

The results of the TOP study will add to the current literature, insofar as it will be the first double-blind randomised comparison of naltrexone and topiramate and will also include a prospective design of a genetic moderator of the topiramate response. The secondary outcomes evaluated in this trial will provide clinically important information about patient groups that are likely to benefit from TOP treatment. The use of an objective laboratory marker of alcohol use is not novel and has been recommended for all alcohol treatment trials [52], but this will be the first topiramate trial to utilise phosphatidylethanol for this purpose. The use of SMS reporting is also an important measure to improve the quality of outcome measurement. Despite it being a self-report measure, SMS reporting occurs very close to real time. It reduces bias and can be used to validate self-reports made at study visits [53]. It is also the first cost-effectiveness analysis of topiramate in the treatment of AUD and the first comparative cost-effectiveness study of topiramate versus naltrexone.

Improving the therapeutic benefit of pharmacotherapy for AUD remains a challenge. The individualised selection of medication based on a patient’s genotype and clinical comorbidities is a potentially cost-saving strategy, as it may avoid the use of futile treatments in individuals unlikely to benefit from them. The TOP trial, thus, has the potential to facilitate the development of a personalised medicine approach and therefore, improve treatment for AUD.

### Trial status

The trial is now in the recruitment phase (commenced 20 June 2017).

## Additional file

Additional file 1: SPIRIT 2013 Checklist: Recommended items to address in a clinical trial protocol and related documents. (DOC 130 kb)
